# Testicular Luxation Secondary to Scrotal Trauma

**DOI:** 10.7759/cureus.83809

**Published:** 2025-05-09

**Authors:** Hernan Aristizabal, Daniel Aristizabal-Mazo, Camilo Giraldo-Villegas

**Affiliations:** 1 Urology Department, Hospital Manuel Uribe Angel, Envigado, COL; 2 Urology Department, Hospital La Maria, Medellin, COL; 3 Urology Department, Universidad de Antioquia, Medellin, COL; 4 General Medicine Department, Universidad Corporación de Estudios de la Salud (CES), Medellin, COL

**Keywords:** case reports, orchiopexy, scrotum, testicular diseases, testis

## Abstract

Testicular luxation is a rare consequence of scrotal trauma, with limited documentation in the literature. We present the case of a 54-year-old patient who presented with a straddle injury following a fall from a horse, resulting in laceration of the left scrotum and complete luxation of the testicle and spermatic cord. The patient required urgent surgical intervention to perform wound debridement, left orchidopexy, and scrotum reconstruction, followed by intravenous antibiotics and postoperative hospital care. This management resulted in preserving the gonadal unit and preventing acute or long-term complications. This case highlights the importance of quick access to urgent urological surgical management for this rare pathology. It emphasizes the importance of subsequent care to avoid potential complications, including testicular loss.

## Introduction

Traumatic luxation of the testicle, or testicular dislocation, is defined as the extra-scrotal migration of one or both testicles [1]. First described in 1818 by Claubry et al., fewer than 200 cases have been described worldwide since then [2,3]. Approximately 80% of cases are caused by motorcycle accidents, primarily in individuals under 20 years of age [4]. Several factors, including the mechanism of trauma, the direction and intensity of impact, the presence of anatomical abnormalities, and the sudden contraction of the cremasteric muscle at the time of injury, influence the site of luxation. The most common site of testicular dislocation is the superficial inguinal area [5]. Due to its low prevalence, this condition presents a limited suspicion rate and is often underdiagnosed. Proper management is directly related to testicular and fertility preservation [6]. The particularity of this case lies in the mechanism of trauma, involving a fall from a horse, which resulted in the complete exposure of the testicle and spermatic cord elements outside the scrotal skin.

## Case presentation

**Figure 1:**
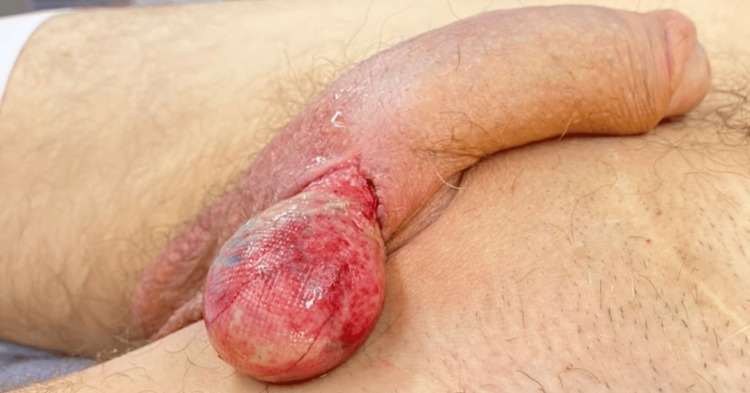
Lateral view Complete externalization of the left testicle

**Figure 2:**
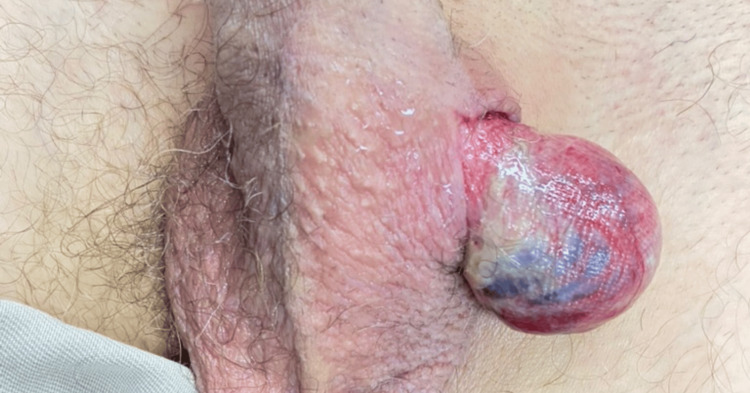
Frontal view Left viable testis

**Figure 3:**
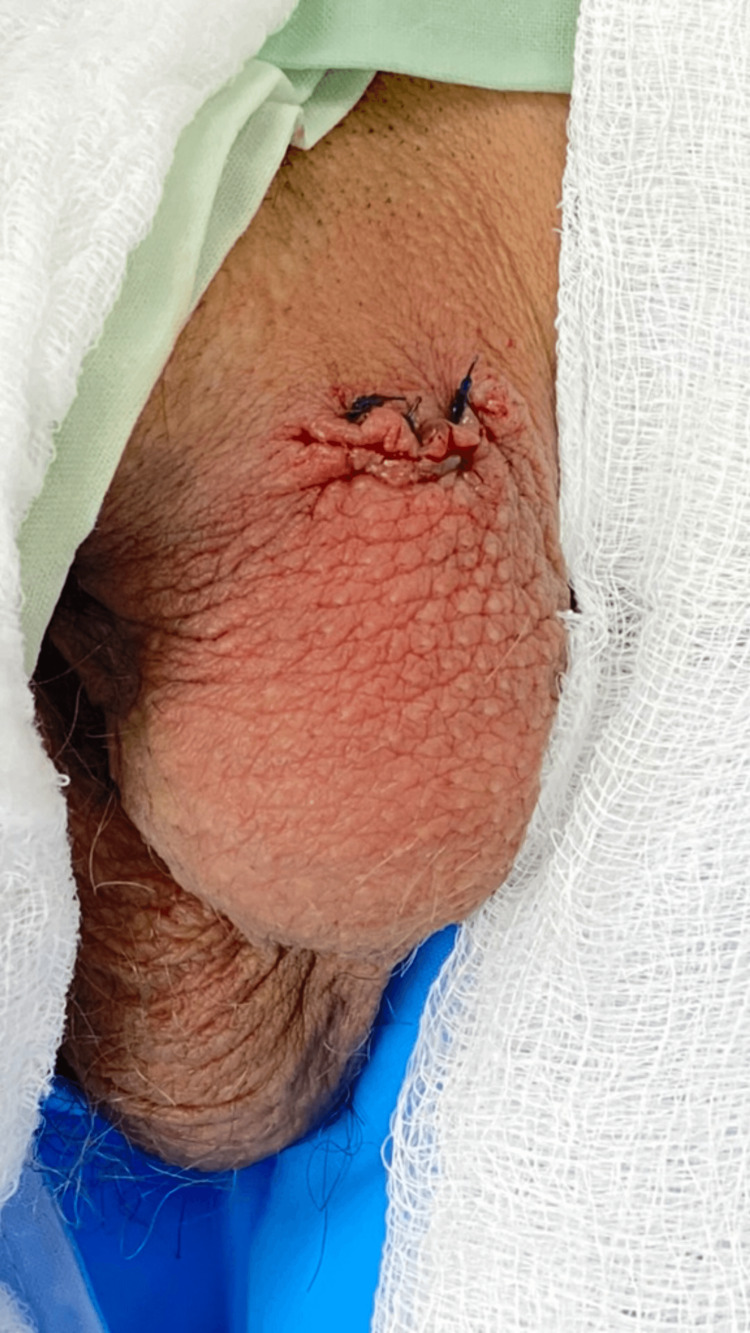
Postoperative reconstruction

We present the case of a 54-year-old male patient with a history of arterial hypertension, type 2 diabetes mellitus, and prior left inguinal herniorrhaphy. The patient sustained a fall from his horse, resulting in blunt straddle trauma to the scrotal region, which caused a wound in the left hemiscrotum through which a mass protruded. Initially, the patient was consulted at a primary care facility, where basic management was provided, and he was subsequently referred to a higher-complexity institution, where the urology team evaluated him. A thorough physical examination revealed an irregular wound located in the superomedial left hemiscrotal area, adjacent to the base of the penis. Complete externalization of the left testicle and spermatic cord through the wound was observed, with apparent gonadal vitality (Figures 1-2). The contralateral testis appeared normal, and no additional signs of trauma were identified in other anatomical regions. The patient was admitted for urgent surgical intervention, and thorough debridement and irrigation of the wound were performed, identifying vital tissue without rupture of the testicle. The testicle was repositioned within the scrotal sac, followed by left orchidopexy and scrotal reconstruction (Figure 3). Postoperatively, the patient was hospitalized for 48 hours, receiving intravenous antibiotic therapy and wound care. He was subsequently discharged without complications. At follow-up, the patient demonstrated satisfactory recovery and healing.

## Discussion

Dislocation, or testicular dislocation, secondary to trauma, is a rare and underreported pathology. No publications are providing conclusive evidence regarding its general management approach. However, a thorough physical examination and clinical suspicion, particularly when correlated with the mechanism of genital trauma, remain the most effective tools for diagnosis, management, and therapeutic decision-making in both surgical and non-surgical contexts [7]. The most extensive review published to date analyzed 45 articles, reporting 105 cases between 1965 and 2021 [3].

In most patients, testicular dislocation is associated with high-energy trauma to the genital and inguinal regions [1]. Motorcycle accidents are the common cause, accounting for more than 80% of reported cases, followed by other types of traffic accidents, which constitute up to 5.7% of cases, and direct multicausal blows, contributing to 3.8% [3]. The displacement and final location of the testicle are influenced by the direction and intensity of the impact, secondary spasmodic contraction of the cremaster muscle, and potential anatomical anomalies such as an inguinal hernia or laxity of the inguinal ring. In this patient, a prior ipsilateral inguinal herniorrhaphy may have played a role in the ectopic displacement of the testis beyond the scrotal skin [5,8].

The most common location of testicular displacement is the superficial inguinal region, accounting for up to 50% of cases. Other reported locations include the pubic region, the base of the penis, the perineal region, the crural region, the canalicular region, and the abdominal cavity. Bilateral dislocations occur in only one-third of cases [9,10]. This case was particularly unusual due to the simultaneous gonadal dislocation and superomedial scrotal rupture. This rupture was caused by the transmission of ascending energy generated by the blunt force of the straddling fall. Remarkably, this mechanism resulted in the complete exposure of the gonad and the spermatic cord without associated penetrating trauma.

Clinically, testicular dislocation without gonadal exposure should be suspected, particularly in young patients with a history of recent or past trauma to the genital or perineal region. A key clinical finding is the absence of the testicle in the scrotal sac. The condition is evident in rare cases, such as the one described here, due to the complete extrusion of testicular contents outside the skin. However, the displacement is often internal and can easily go unnoticed [9].

On physical examination, Brockman’s sign (characterized by an empty, well-developed scrotal sac with lax skin) is a classic indicator. This finding is often accompanied by the palpation of an oval mass in the inguinal canal or surrounding tissues. However, intense pain, localized edema, scrotal hematoma, or hematocele can frequently delay or obscure the diagnosis during initial evaluation. Direct visualization of the testicle through the scrotal skin immediately identifies the pathology [5,6,11].

When the diagnosis is uncertain or significant local inflammatory changes are present, Doppler ultrasound can help confirm the location of the testicle and assess vascularization. It is utilized in up to 21.9% of cases. In selected cases, particularly in polytraumatized patients, contrast-enhanced CT of the abdomen and pelvis may also be employed for further evaluation [3,5].

Surgical management is the treatment of choice. Although some authors recommend manual reduction in acute diagnoses to prevent complications, orchidopexy remains the definitive treatment for these patients. In cases involving genital wounds, thorough intraoperative irrigation with sterile solutions, debridement of devitalized tissue, and scrotal reconstruction in conjunction with testicular fixation are the standard approaches [4,5,9,12].

This type of trauma requires urgent intervention, with the primary objective being the preservation of testicular integrity and function. If treatment is delayed, complications such as necrosis, infections (including abscess formation), fertility impairment, hypogonadism, and multifactorial chronic pain syndrome may appear [13,14].

## Conclusions

Testicular luxation, or dislocation, although rare, should not be considered exceptional in the context of genital trauma. An appropriate clinical approach, including a detailed physical examination of the scrotum and genital area, is essential for accurate diagnosis. Diagnostic tools such as testicular Doppler ultrasound and, in selected cases, contrast-enhanced CT allow characterization of the lesion in case of uncertainty. Reducing the interval between trauma and definitive surgical treatment is critical to minimizing complications, with an emphasis on testicular preservation as the primary objective.
